# Genetic modifiers of upper limb function in Duchenne muscular dystrophy

**DOI:** 10.1007/s00415-022-11133-8

**Published:** 2022-05-05

**Authors:** Daniele Sabbatini, Aurora Fusto, Sara Vianello, Matteo Villa, Joanna Janik, Grazia D’Angelo, Eleonora Diella, Francesca Magri, Giacomo P. Comi, Chiara Panicucci, Claudio Bruno, Adele D’Amico, Enrico Bertini, Guja Astrea, Roberta Battini, Luisa Politano, Riccardo Masson, Giovanni Baranello, Stefano C. Previtali, Sonia Messina, Gianluca Vita, Angela Berardinelli, Tiziana Mongini, Antonella Pini, Marika Pane, Eugenio Mercuri, Eric P. Hoffman, Lauren Morgenroth, Heather Gordish-Dressman, Tina Duong, Craig M. McDonald, Luca Bello, Elena Pegoraro

**Affiliations:** 1 Department of Neurosciences DNS, University of Padova, via Giustiniani, 5, 35128 Padua, Italy; 2 Scientific Institute IRCCS E. Medea, NeuroMuscular Unit, Lecco, Bosisio Parini Italy; 3 IRCSS Foundation, Ca’ Granda Ospedale Maggiore Policlinico; Dino Ferrari Centre, Department of Pathophysiology and Transplantation (DEPT), University of Milan, Milan, Italy; 4 Center of Translational and Experimental Myology, IRCCS Istituto Giannina Gaslini, and Department of Neuroscience, Rehabilitation, Ophtalmology, Genetics, Maternal and Child Health–DINOGMI,University of Genoa, Genoa, Italy Genoa Italy; 5 Unit of Neuromuscular and Neurodegenerative Disorders, Bambino Gesù Children’s Hospital IRCCS, Rome, Italy; 6 Department of Developmental Neuroscience, IRCCS Stella Maris, Calambrone, Pisa Italy; 7 Cardiomiology and Medical Genetics, Department of Experimental Medicine, “Vanvitelli” University of Campania, Naples, Italy; 8 Developmental Neurology Unit, Fondazione IRCCS Istituto Neurologico Carlo Besta, Milan, Italy; 9 The Dubowitz Neuromuscular Centre, NIHR BRC University College London Great Ormond Street Institute of Child Health & Great Ormond Street Hospital, London, UK; 10 Neuromuscular Repair Unit, Inspe and Division of Neuroscience, IRCSS San Raffaele Scientific Institute, Milan, Italy; 11 Department of Neurosciences and Nemo Sud Clinical Center, University of Messina, Messina, Italy; 12 C. Mondino Foundation, Pavia, Italy; 13 Neuromuscular Center, AOU Città Della Salute E Della Scienza, University of Torino, Turin, Italy; 14 Pediatric Neuromuscular Unit, IRCCS Istituto Delle Scienze Neurologiche Di Bologna, Bologna, Italy; 15 Pediatric Neurology, Department of Woman and Child Health and Public Health, Università Cattolica del Sacro Cuore, Child Health Area, Rome, Italy; 16 Centro Clinico Nemo, Fondazione Policlinico Universitario Agostino Gemelli IRCCS, Rome, Italy; 17 Binghamton University - SUNY, Binghamton, NY USA; 18 Center for Genetic Medicine, Children’s Research Institute, Children’s National Health System, Washington, DC USA; 19 Department of Neurology, Stanford University School of Medicine, Palo Alto, CA USA; 20 University of California Davis Medical Center, Sacramento, CA USA

**Keywords:** Duchenne muscular dystrophy, Genetic modifiers, Upper limb function, SPP1–osteopontin, CD40

## Abstract

Genetic modifiers of Duchenne muscular dystrophy (DMD) are variants located in genes different from the disease-causing gene *DMD*, but associated with differences in disease onset, progression, or response to treatment. Modifiers described so far have been tested mainly for associations with ambulatory function, while their effect on upper limb function, which is especially relevant for quality of life and independence in non-ambulatory patients, is unknown. We tested genotypes at several known modifier loci (*SPP1*, *LTBP4*, *CD40*, *ACTN3*) for association with Performance Upper Limb version 1.2 score in an Italian multicenter cohort, and with Brooke scale score in the Cooperative International Neuromuscular Group Duchenne Natural History Study (CINRG-DNHS), using generalized estimating equation (GEE) models of longitudinally collected data, with age and glucocorticoid treatment as covariates. *CD40* rs1883832, previously linked to earlier loss of ambulation, emerged as a modifier of upper limb function, negatively affecting shoulder and distal domains of PUL (*p* = 0.023 and 0.018, respectively) in the Italian cohort, as well as of Brooke score (*p* = 0.018) in the CINRG-DNHS. These findings will be useful for the design and interpretation of clinical trials in DMD, especially for non-ambulatory populations.

**Supplementary Information:**

The online version contains supplementary material available at 10.1007/s00415-022-11133-8.

## Introduction

Duchenne muscular dystrophy (DMD) is a severe and progressive muscle disease caused by complete dystrophin deficiency in muscle fibers. It is an X-linked recessive disease, with an incidence of around 1 in 3800–4200 male births and prevalence between 19.9 and 95.5 in 1,000,000. Usually, symptoms are present in early childhood with delayed motor milestones and difficulties in rising from the floor, typically with a Gowers’ manoeuver, and in climbing stairs. Progressive muscle degeneration causes loss of independent ambulation (LoA) typically around the age of 13. Respiratory and cardiac involvement develop later, and are major causes of death [1].

Even if all DMD patients carry out-of-frame mutations that disrupt protein expression completely, still it is possible to observe a spectrum of phenotype severity within DMD [2–5]. This is primarily measured by age at LoA, because of its impact on daily life and the overall health of patients, and its correlation with overall survival and other disease milestones, such as the onset of respiratory insufficiency and the need for scoliosis surgery [6]. All of these disease milestones may vary by several years, e.g. loss of ambulation may ensue from before 10 years to after 15 years of age.

Phenotype variability in DMD may be caused by environmental (e.g. socioeconomic conditions, treatments) and genetic effects. The genetic effects can be further subdivided in “*cis*” and “*trans*” acting effects. The former ones are due to the *DMD* mutations themselves: in fact, in DMD patients, dystrophin may not always be completely absent from skeletal muscle fibers. Protein assays that are commonly used in the diagnostic setting have limited sensitivity, so that small amounts of protein may escape detection, while still exerting a measurable effect on the phenotype [6]. The “*trans*” acting factors are genetic modifiers, i.e. polymorphisms in genes different than *DMD*, which influence disease phenotype, affecting onset, progression, response to treatment, etc. Several loci have been shown to modify LoA in DMD: *SPP1* rs28357094 [7], *LTBP4* rs10880, rs2303729 and rs1131620 [8], *CD40* rs1883832 [9], *ACTN3* rs1815739 [10], *THBS1* rs2725797 and rs2624259 [11]. All these genes are involved in key features of DMD pathogenesis, such as inflammation, fibrosis, response to treatment, and muscle function [6, 12].

In the non-ambulatory stages of DMD, upper limb function obviously suffers a progressive decline, which strongly influences patient independence and quality of life. Therefore, dedicated outcome measures have been specifically developed, including the Brooke score [13] and the Performance Upper Limb (PUL) scale [14]. Here, we aimed to verify if genetic modifiers of LoA in DMD, both *cis*- and *trans*-acting, also affect the performance of the upper limbs measured with the PUL test. Moreover, all associations were tested for validation in an independent cohort (i.e. Cooperative International Neuromuscular Research Group Duchenne Natural history Study, CINRG-DNHS) in which patients had been tested using the Brooke scale. PUL was not available in the CINRG-DNHS, as the scale had not yet been designed at the time the DNHS protocol was finalized.

## Methods

### Patient selection

Retrospective data were collected from several Italian Centers (i.e. University of Padova, University of Milan, “Mondino” Institute in Pavia, IRCCS “Medea” in Bosisio Parini, “Besta” Neurological Institute in Milan, University of Turin, “Gaslini” Institute in Genova, IRCCS “Bellaria” in Bologna, IRCCS “Stella Maris” Pisa, “Bambin Gesù” Hospital in Rome, Catholic University of the Sacred Heart in Rome, University “Vanvitelli” in Naples, NEuroMuscular Omnicenter NEMO in Messina). Inclusion criteria were: molecularly confirmed DMD diagnosis, at least one available PUL evaluation, and the availability of genomic DNA for SNPs genotyping. Exclusion criteria were: an in-frame *DMD* mutation and/or preserved dystrophin expression in the muscle biopsy; inability to carry out a reliable PUL test as ascertained by trained evaluators. Patients from the Cooperative International Neuromuscular Research Group Duchenne Natural History Study (CINRG-DNHS) were used as validation cohort; inclusion criteria and cohort characteristics have been previously described [15].

### *DMD* genotype

Information about pathogenetic *DMD* mutations was collected when available from clinical records or genetic reports. Patients were clustered, according to *DMD* gene mutations and their amenability molecular treatments, i.e. skipping of exons 8, 44, 45, 51, and 53 (henceforth: “skip 8”, “skip 44”, etc.), duplications, splice site mutations, and nonsense mutations. Rarer deletions were clustered in the “other deletions” subset. Missense mutations were not present in our population.

### PUL test

The PUL scale version 1.2 was used to evaluate the performance of upper limbs in the Italian cohort as previously described by Mayhew and colleagues [16]. The test is composed of 22 items, 21 of which assess the functionality of upper limbs, divided in 3 domains: proximal domain (henceforth “Shoulder”), medial domain (henceforth “Elbow”), and distal domain (henceforth “Distal”). The first item (item A) allows to evaluate overall proximal function, and is very similar to the Brooke scale (supplementary table 1 for Brooke and PUL scale comparison). Total PUL score is calculated by the sum of all items, excluded item A.

### Brooke scale

To assess upper limb function in the validation cohort, the Brooke scores was used. On the Brooke scale (range from 1 to 6), a score equal to 1 is considered when the patient is able to start with arms at the sides and can abduct the arms in a full circle until they touch above the head, while 6 means they have no useful hand function [13]. All functional assessments were performed by trained physiotherapists.

### Targeted genotyping

Patients’ DNA samples were genotyped, using TaqMan (Thermo Fisher Scientific) assays, at these known DMD modifier loci: *SPP1* rs28357094 [7], *LTBP4* rs10880, rs2303729 and rs1131620 [8], *CD40* rs1883832 [9], *ACTN3* rs1815739 [10]. For tests of genotype/phenotype association, we used the same inheritance models as in published reports. Allele frequencies were tested for Hardy–Weinberg equilibrium.

### Statistical analyses

Quantitative variables were summarized as mean ± standard deviation (SD) and median (range), unless otherwise specified. Intervals of linear decrease of PUL scores (Total PUL, Shoulder, Elbow, and Distal) measures were defined on the age axis by piecewise regression, using baseline data (i.e. earliest available value) and choosing a 1-break model for total PUL, Elbow and Distal, and linear model without break point for Shoulder after visual inspection of the scatter plot. Generalized Estimating Equations (GEEs) were used to estimate effects of: age, GC treatment (on vs. off at each evaluation), *DMD* mutation, and SNP genotypes (dominant, recessive, or additive as appropriate). GEEs were applied within the “linear” age range defined by piecewise regression. The validation cohort was tested using GEE models to analyze the effect of SNPs genotype, age, and GC treatment (considered "true" when patients have taken them for at least one year in their life) on the Brooke score. Statistical analyses were performed with R v.4.0.2.

## Results

### PUL cohort

The Italian multi-centric cohort was composed of 137 patients. The average age was 11.38 ± 5.22 (minimum age: 4.17, maximum: 28.59). During follow-up, 88 patients (64.2%) were continuously on GCs, 32 (23.4%) were never on GCs, 15 (10.9%) switched on/off (5 patients started, 10 stopped) and 2 (1.5%) had no available GC treatment data. There were significant differences in age between GC treatment subgroups (Kruskal–Wallis test *p* < 0.001). Patients who were continuously on GCs were younger (10.14 ± 4.05, range 4.17–24.75 years) than those continuously off GCs (15.98 ± 6.19, range 4.83–28.59 years), and patients who started GCs during follow-up were the youngest subgroup (6.99 ± 2.81, range 4.31–11.61 years).

### PUL results

A total of 636 PUL assessments were obtained from the 137 patients, 125 of whom had more than one PUL evaluation available, with a mean of 4.64 evaluations per patient (range 1–15 PULs/patient). Mean ± standard deviation of follow-up duration was 2.82 ± 1.30 years (range 0.98–5.70 years). Mean age at first evaluation was 11.38 ± 5.22 years (range 4.17 sec228.59 years), while at last evaluation it was 14.16 ± 5.26 years (range 5.69–29.67 years).

Total and domain-specific scores at baseline increased until approximately 6–8 years of age, and then decreased progressively (as expected). We used the piecewise regression model to estimate the ranges of linear decrease of total PUL and sub-domains scores. We found that total PUL total score decreases in a linear fashion starting from age of 7.5, shoulder score from 6.7, elbow from 8.9, and distal from 8.7 years of age. Stratifying patients based on GC treatment, treated patients had higher PUL scores compared to non-treated, although there was a decreasing trend with age in both treated and untreated patients. Regarding total PUL scores, it appears that the rate of decline is similar between treated and untreated groups, but with higher values for treated, likely because of a higher and longer plateau of maximum function. The domain which seems to differentiate most between treated and untreated subgroups is the elbow domain, while shoulder and distal domain appear less differentiated.

### Brooke results

A total of 2895 evaluations were obtained with Brooke test from 340 patients of CINRG-DNHS cohort. Two hundred and eighty of them had more than one evaluation available, with a mean Brooke score at baseline. The mean follow-up time was 5.6 ± 2.41 years (range 0.23–9.9 years). The mean age at the first evaluation was 11.98 ± 5.8 years (range 2.05–28.01 years), while at the last evaluation, it was 17.59 ± 5.78 years (range 4.5–33.85 years).

### Genotyping results

Patients from the two cohorts were genotyped for *SPP1* rs28357094, *CD40* rs1883832, *LTBP4* rs2303729, rs1131620 and rs10880, and *ACTN3* rs1815739. In both cohorts, there were patients for whom it was not possible to assess Genotype at all the SNPs, in particular: *CD40* rs1883832 data were missing for 2 patients from the Italian and 63 from the CINRG cohort, *LTBP4* rs1131620 data were missing for 1 patient belonging to the Italian cohort and 82 from the CINRG cohort, *LTBP4* rs10880 genotype was not available for 3 patients from the Italian and 66 from the CINRG cohort, *LTBP4* rs2303729 was missing for 75 CINRG patients, *SPP1* rs28357094 data were missing for 61 patients from the CINRG cohort, and it was not possible to assess the genotype at *ACTN3* rs1815739 for 1 Italian and 75 CINRG patients. All the genotypes did not deviate from the Hardy–Weinberg equilibrium, and the observed MAFs were compatible with those expected from the European population.

### GEE models

To determine if the genotypes at the modifier loci, together with patient’s age and GCs treatment, influence the PUL scores (total, shoulder, elbow and distal) in the Italian cohort or Brooke score in CINRG-DNHS, we used the GEE model. Results are summarized in Tables 1 and 2.

**Table 1:** Estimates of effects of several variables on longitudinal PUL measures, based on GEE models, in patients with age ≥ 7.51 years in the Italian cohort

Parameter	PUL		Shoulder		Elbow		Distal	
	Estimate	*p* value	Estimate	*p* value	Estimate	*p* value	Estimate	*p* value
Intercept	78.49	< 0.001	20.61	< 0.001	33.60	< 0.001	23.51	< 0.001
Age	– 1.97	< 0.001	– 0.91	< 0.001	– 0.82	< 0.001	– 0.16	< 0.001
GC	11.19	< 0.001	1.15	n.s	9.35	< 0.001	2.08	< 0.001
*SPP1* rs28357094 dom	– 1.49	n.s	– 0.49	n.s	– 1.71	n.s	– 0.12	n.s
*CD40* rs1883832 add	– 3.07	0.076	– 1.55	0.023	– 1.11	n.s	– 0.65	0.018
*ACTN3* rs1815739 add	– 2.20	n.s	– 0.63	n.s	– 2.09	0.030	– 0.61	0.025
*LTBP4* rs10880 rec	– 0.12	n.s	– 1.13	n.s	0.83	n.s	– 0.06	n.s

*PUL* performance of upper limbs, *GC* glucocorticoid treatment. The models adopted for SNPs are dominant (dom), recessive (rec) or additive (add)

**Table 2:** Estimates and *p* values for two-tailed tests for SNPs researched on a subset of the CINRG-DNHS cohort

Parameter	Brooke	
	Estimate	*p* value
Intercept	– 1.61	< 0.001
Age	0.21	< 0.001
GC	0.95	< 0.001
*SPP1* rs28357094 dom	0.28	0.034
*CD40* rs1883832 add	0.29	0.018
*ACTN3* rs1815739 add	0.21	0.2
*LTBP4* rs10880 rec	– 0.30	0.1

These models estimated linear coefficients of yearly decrease for the total score and domain sub-scores, which are similar, but slightly lower than those estimated by simple linear regression using baseline scores only. This result may be expected based on the statistical features of the models.

In the Italian cohort, coefficients relative to GC treatment status at the time of each PUL evaluations corresponded to + 11.19 points in the total score (*p* < 0.001), + 1.15 points in the shoulder sub-score (*p* = n.s.), + 9.35 points in the elbow subscore (*p* < 0.001), and + 2.08 points in the distal subscore (*p* < 0.001). Notably, coefficients relative to GC treatment here presented are not dependent on grouping of individual patients based on treatment status, but refer to GC treatment as a dichotomic variant (on vs. off) at each PUL evaluation included in the model.

**Fig. 1:**
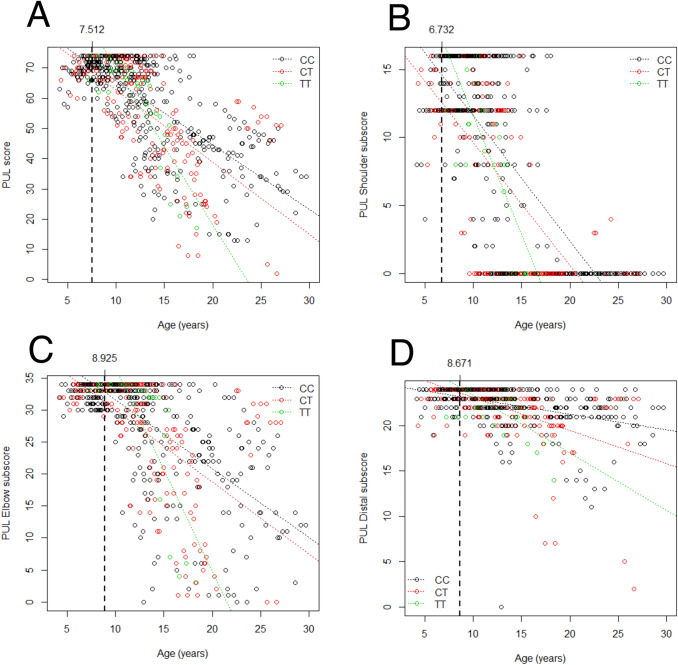
Scatter plots of PUL values grouped by *CD40* rs1883832 genotype. Individual data points (which may include multiple data points from individual patients) are color-coded based on genotype, and a regression line for each genotype group is plotted. The vertical dashed lines, annotated on top with the corresponding value on the *x* (i.e. age) axis, indicate the age at which the piecewise regression models predicts the beginning of a linear decline of the measure. Panel A shows total PUL scores, while panels B, C, and D show subscores for the shoulder, elbow, and wrist domains, respectively

**Fig. 2:**
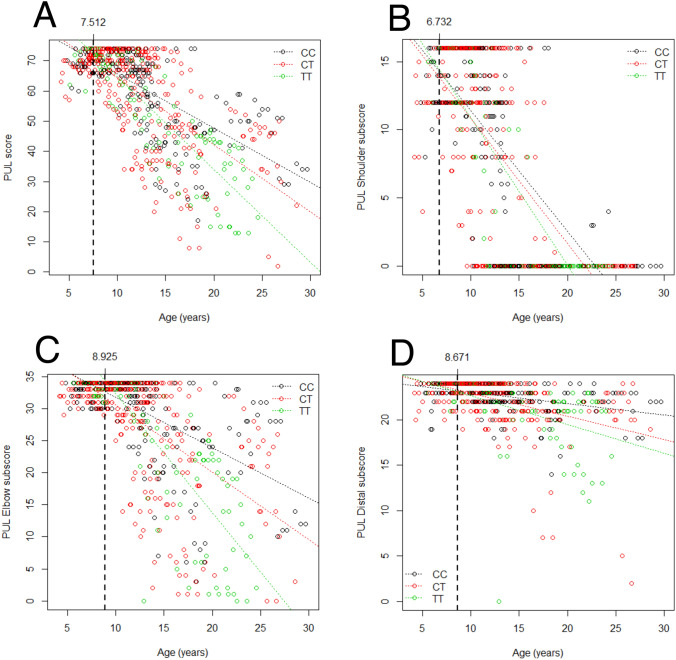
Scatter plots of PUL values grouped by *ACTN3* rs1815739 genotype. The T genotype corresponds to a null *ACTN3* allele (nonsense SNP). Individual data points (which may include multiple data points from individual patients) are colored based on genotype (see legend), and a regression line for each genotype group is plotted. The vertical dashed lines, annotated on top with the corresponding value on the *x* (i.e. age) axis, indicate the age at which the piecewise regression models predicts the beginning of a linear decline of the measure. Panel A shows total PUL scores, while panels B, C, and D show subscores for the shoulder, elbow, and wrist domains, respectively

The inheritance models used for SNPs were based on those found in the literature (see Methods). In regard to *LTBP4*, here we present results relative to the isolated rs10880 genotype (the most strongly associated with phenotypes in published literature), but alternative models for the IAAM homozygous haplotype were also explored with similar findings (data not presented). In a multivariate GEE model that evaluated concurrent effects of all 4 SNPs, age, and GC treatment, significant associations were observed between additive *CD40* rs1883832 genotype and shoulder/distal PUL subscores (detrimental effect of T genotype, *p* = 0.023 and 0.018 respectively), with a trend of association in the total score (*p* = 0.076). Additive *ACTN3* rs1815739 genotype was also significantly correlated with elbow and distal subscores (lower scores with the null allele, *p* = 0.030 and 0.025 respectively) (Table 1). Scatter plots of PUL scores by *CD40* and *ACTN3* genotypes are shown in Figs. 1 and 2, respectively.

Using a multivariate GEE model, it was possible to establish the concurrent effects of *SPP1* rs28357094, *CD40* rs1883832, *LTBP4* rs10880 SNPs, *ACTN3* rs1815739, age, and GC treatment on Brooke score in the CINRG-DNHS cohort. The coefficients relative to GC treatment status at the time of each evaluations corresponded to + 0.95 points (*p* < 0.001), for the Brooke score. Moreover, it was possible to assess a significant association of *CD40* rs1883832 (additive model) and *SPP1* rs28357094 (dominant) with the Brooke score.

### Mutation analyses

*DMD* gene mutations were fully characterized for 96 patients from the Italian cohort and were divided as follows: 64 (64/96, 66.7%) were deletions of one or more exons; 9 (9/96, 9.4%) duplications of one or more exons, and the remaining 23 (23/96, 23.9%) were small intraexonic or intronic mutations.

The mutations were divided in groups based on their eligibility for exon skipping or other molecular treatments (see Methods). The remaining mutations were divided into duplications, nonsense mutations, splice site mutations, and other deletions (when not classifiable to other groups).

Significant correlation was found for the skip 45, skip 8 and skip 53 and skip 51 groups. The skip 45 group was positively correlated (beneficial effect) with total PUL scores (*p* = 0.042) and shoulder subscores (*p* = 0.002), and with distal subscores (*p* = 0.017), but not with elbow subscores. The skip 53 and skip 51 groups were negatively correlated (detrimental effect) with PUL scores, but only at the elbow level (*p* < 0.001 and 0.012, respectively) level. The skip 8 group was found to be positively correlated with the PUL scores with regard to total PUL scores (*p* = 0.015), shoulder level (*p* < 0.001), elbow level (0.037), and distal level (*p* = 0.002). It is necessary to note that the skip 8 and skip 45 groups were small (skip 8 = 3 patients, skip 45 = 5 patients), with relatively young patients and little variability in age. Concerning the CINRG cohort, *DMD* gene mutations were fully characterized for 287 patients and were divided as follows: 208 (208/287, 72.5%) were deletions of one or more exons; 14 (14/287, 4.8%) duplication of one or more exons; and the remaining 65 (65/287, 22.6%) were small intraexonic or intronic mutations. None of these mutation groups was significantly associated with Brooke scores Table 3.

**Table 3:**
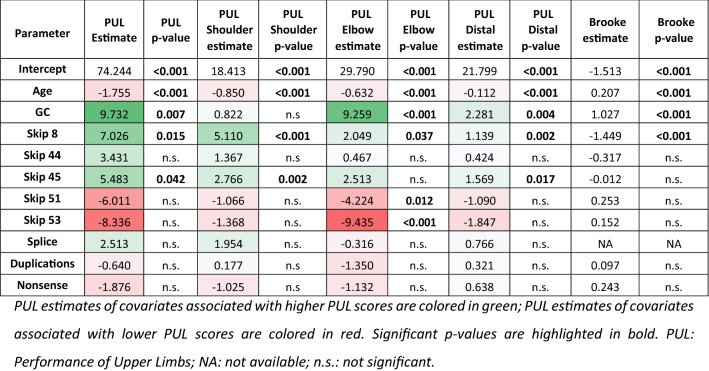
Estimates and p values for the groups of mutations considered in the Italian and CINRG cohorts

PUL estimates of covariates associated with higher PUL scores are colored in green; PUL estimates of covariates associated with lower PUL scores are colored in red. Significant *p* values are highlighted in bold. *PUL* performance of upper limbs, *NA* not available, *n.s.* not significant

## Discussion

The primary goal of this work was to describe the clinical course of the disease in our population based on the PUL score. We performed a piecewise regression on the baseline data finding a breakpoint at 7.512 years. Considering the clinical history of the disease, this finding was not surprising, as patients with DMD early on improve their motor abilities. In fact, also for ambulation-related phenotypes (6MWT, NSAA) it is usually possible to observe a plateau phase around 7 years of age [17], and only then, with the progression of the disease, motor function worsens. It is relevant that the age of the plateau of upper limb function was similar to that of ambulatory function, although the latter is commonly believed to deteriorate earlier. Even if the affection of the upper limbs is clinically noticeable only later in the disease, it is reasonable to expect this finding. We suggest that the decrease in score at this age would be mostly caused by impairment of function at shoulder level, which is the first level of upper limb to be affected in DMD. Indeed, when we performed the piecewise regression on the partial scores, breakpoints were earlier at the shoulder level (6.732 years of age), than at the elbow and distal levels (8.925 years and 8.671 years). The shoulder level is indeed the first to become altered, in clinical history. What seemed unusual was to find a breakpoint at distal level to be a little earlier than the breakpoint at the elbow level since the distal level is the last to be significantly impaired. This discrepancy could be due to the small number of patients in our population, to the slower decrease of the distal subscore, or to effects of GC treatment. Another important finding in this study was the estimated yearly decrease of PUL score (approximately 2 points/year, see Table 1), representing a combination of points lost at all three levels of PUL. The larger part of this loss is attributable to the shoulder and elbow levels, as one would expect based on the clinical course of the disease. The distal level, as expected, has a small role (0.3761 points) in the total points lost in a year, as this region is the last to lose its motor function. Importantly, one has to consider the limits of ordinal data such as clinical scale scores, when linear methods are applied. As a general rule, linear methods can be reliably applied to ordinal data if the scale has a sufficiently large number of levels (as is the case of PUL with 74 levels) and sufficient reliability.

A positive correlation of GC treatment on PUL performance was confirmed, but there was an unexpected finding. Considering both the SNPs and the mutational group, the association with GC that was extremely significant when considering total PUL scores (*p* < 0.001, *p* = 0.01), became non-significant when considering only the partial scores of the shoulder. This anomaly could be caused by the fact that the shoulder level starts to be affected early on, when children are young and are not yet under GC treatment. The significance is then again high when considering the other two levels of PUL. This can be interpreted as a limit of an observational, retrospective study, in which patients are not randomized to treatment.

Among the genetic modifiers we considered, only *SPP1* had been previously validated using grip strength (i.e. upper limb muscle strength) [7], and thus it would have been reasonable to expect similar results using the PUL and Brooke scale. However, we found a significant association between the G allele at the promoter of *SPP1* and upper limb strength only in the CINRG cohort. Possible explanations of the lack of significance in the Italian population may include a relatively small effect of the allele, and the relatively low proportion of patients on continuous GC treatment (64%) in this cohort, since it has been proposed that *SPP1* genotype acts as a modulator of GC response [18].

The second genetic modifier analyzed was rs1815739 in *ACTN3*. This SNP predicts a null polymorphism, thus homozygous individuals show absence of α-actinin-3 in muscle. The loss of α-actinin-3 has a complex effect in muscle, decreasing strength but supposedly ameliorating the clinical course in the long term, probably because of a shift from fast-type to slow-type muscle fibers, the latter being relatively spared in DMD. Hogarth et al. showed an effect of rs1815739 on LoA, 6MWT, and strength of upper and lower limbs, however the effect on LoA did not reach statistical significance [10]. In our study, while there were no significant associations with PUL in general, nor with Brooke performances, we did find significant associations with elbow and distal PUL scores, in the same direction (i.e. detrimental) as earlier LoA and reduced grip strength previously reported [10]. It is reasonable that reduced strength associated with α-actinin-3 may reduce ability in PUL items which require maximal efforts (e.g. lifting heavy weights or tearing folded paper). Furthermore, muscles in the arms, forearms, and hands, which are known to contain a high ratio of type II glycolytic fibers where this isoform of actinin is mostly expressed, may be more affected than proximal, larger muscles, which have a higher proportion of type I fibers. Our findings may represent an indirect validation of the modifier effect of *ACTN3*, using phenotypes that are not identical but correlated to the same underlying variable, i.e. muscle strength.

The third genetic modifier considered in this study is rs1883832 in *CD40,* that showed the strongest association with PUL and Brooke scores. The direction of the effect was negative for the minor T allele, in concordance with described effects on LoA. The estimated linear coefficients of yearly decrease showed a higher impact at the shoulder level than at the distal level. The lesser effect at distal level seems plausible considering the progression of DMD, in which the distal level is the last and least affected. The effect of rs1883832 has been confirmed also in the validation cohort (*p* = 0.018 for the Brooke scale). The putative mechanism of action of *CD40* minor allele “T” could be in decreasing muscle regenerative ability and increasing fibrosis. In fact, the transition from innate to adaptive immunity, in which CD40 is implicated, is relevant in muscular dystrophy, especially with regards to the balance between macrophages with pro-fibrotic (M1) vs pro-regenerative (M2) phenotype [9, 19].

The last examined genetic modifier was *LTBP4*, for which we failed to find significant association between PUL scores and all the SNPs in *LTBP4* genotypes. The same result was obtained with the validation cohort. This was somewhat surprising, given that *LTBP4* appears to be one of the modifiers with the largest effect size, and most consistently validated across international cohorts [8, 18, 20].

As mentioned previously, the phenotypic variability that can be observed in DMD patients can be partially explained by different types of *DMD* mutations. For these reasons, we chose to select a few specific groups of mutations, as analyzing each single mutation would have led to an excessive fragmentation of the cohort, barring statistically significant conclusions. The effect of different mutations on age at LoA has been reported by several recent studies. The greater effect was seen in patients amenable to exon skipping of exons 44 [21, 22], 8 [5, 18] and 51 [23], where those eligible to skipping of exon 44 and 8 had a milder phenotype with a delay in age at LoA and patients amenable to skipping of exon 51 had a poorer outcome. Patients amenable to exon skipping of exon 53 had also been found to be associated with a different clinical course than other mutations. Servais et al. reported a greater severity linked to this phenotype, with lower left ventricular ejection, more severe contractures, reduced strength in upper limbs, and earlier age at LoA relatively to other theoretical skip groups considered [24].

In our study, a significant association between “skip44” group and PUL scores was not confirmed, despite the ample evidence in the literature of an effect on ambulatory phenotypes. We have found instead a significant association between the “skip45” group and the PUL scores, with a favorable effect on the function of upper limb considering the total PUL scores (*p* = 0.042), the shoulder level (*p* = 0.002) and the distal level (*p* = 0.017). The significance was not found for elbow level. There is no obvious rational explanation for this difference. Considering the small number of patients in this group and their relatively young age, these results may be a result of bias, and therefore should be validated in a larger cohort before this association can be established. Statistically significant reductions of PUL scores were found in the “skip53” group, already reported to have poorer prognosis [24]. However, in our population, statistical significance was found only at the elbow level (*p* < 0.001). Comparable results were found for “skip51” subgroup, with statistically significant reductions of PUL subscores at elbow level (*p* = 0.012). A poorer prognosis in “skip51” subgroup has been already reported by Wang et al. [5] As both the patients in “skip51” and “skip53” subgroups are deficient of the Dp140 dystrophin isoform, which is largely expressed in the brain, and have higher incidence of cognitive issues [25, 26], it may be that executive function issues impair PUL scores to some extent. Although small, the “skip8” group was the one that we found to have the most significant association with PUL total score (*p* = 0.02). As reported in the literature [22, 27–29] for overall and ambulatory phenotypes [22, 27–29], this group had a milder course, with slower decrease in PUL score. This effect was present not only on total PUL score, but also at the shoulder level (*p* < 0.001) and elbow level (*p* = 0.04). This group in our population was comprised of only 3 patients, and their age distribution was skewed toward the younger age, and this could theoretically bias our results. However, the effect seems expected based on literature data, and is probably genuine. In fact, our group was comprised of three patients, two still ambulating and one with age at LoA of 16.1 years, which is higher than the average age of LoA.

In conclusion, we describe the natural history of upper limb dysfunction in DMD by identifying ages of maximum function before the start of deterioration at the global, shoulder, elbow, and distal levels. We confirmed that shoulder function is lost earlier, and quantified yearly decline rates of PUL scores. We identified significant effects of *CD40* genotype on shoulder and distal function (the rs1883832 T allele being detrimental), and of *ACTN3* on elbow and distal function considering the Italian cohort, and confirmed the detrimental effect of the G allele in *SPP1* rs28357094 allele in the validation cohort. Deletions amenable to exon 44 skipping did not show clearly preserved upper limb function, which was clear with deletions amenable to exon 8 skipping. Deletions eligible for exon 53 and 51 skipping, on the other hand, showed worse impairment of elbow function than average DMD. All these findings will be useful in designing and interpreting clinical trials in DMD, especially when targeting populations across the ambulatory and non-ambulatory range; moreover, identified genetic modifiers may be considered as potential therapeutic targets.

## Supplementary Information

Below is the link to the electronic supplementary material.

Supplementary file1 (DOCX 28 KB)
