# Supplementary material for: Genetic modifiers of upper limb function in Duchenne muscular dystrophy
Source: J Neurol. 2022 May 5;269(9):4884–94. doi: 10.1007/s00415-022-11133-8 (PMC9363325; doi:10.1007/s00415-022-11133-8)
Supplement: Supplementary file 1 — Supplementary file1 (DOCX 28 KB) [file 415_2022_11133_MOESM1_ESM.docx]

SUPPLEMENTARY

Supplementary Table 1. Comparison of PUL and Brooke scales.

| **Score description** | **PUL Scale** | **Brooke Score** | **Score description** |
| --- | --- | --- | --- |
| Can abduct both arms simultaneously elbows in extension in full circle until they touch above the head | 6 | 1 | Starting with arms at the sides, the patient can abduct the arms in a full circle until they touch |
| Can raise both arms simultaneously above head only by flexing the elbow (shortening circumference of the movement/using accessory muscles) | 5 | 2 | Can raise arms above head only by flexing the elbow (shortening the circumference of the movement) or using accessory muscle |
| Can raise both arms (to shoulder height with or without compensation) | 4 | 3 | Cannot raise hands above head, but can raise a 8-oz glass of water to the mouth |
| Can raise plastic cup with 200g weight in it to mouth using 1 or 2 hands | 3 |  |  |
| Can raise 1 or 2 hands to mouth but cannot raise a cup with a 200g weight in it to mouth | 2 | 4 | Can raise hands to the mouth, but cannot raise a 8-oz glass of water to the mouth |
| Can use hands to hold pen or pick up a coin or drive a powered chair | 1 | 5 | Cannot raise hands to the mouth, but can use hands to hold a pen or pick up pennies from the table |
| No useful function of hands | 0 | 6 | Cannot raise hands to the mouth and has no useful function of the hands |
